# A B-Box (BBX) Transcription Factor from Cucumber, CsCOL9 Positively Regulates Resistance of Host Plant to *Bemisia tabaci*

**DOI:** 10.3390/ijms26010324

**Published:** 2025-01-02

**Authors:** Shuixiang Xie, Baozheng Shi, Mengzhen Miao, Chenchen Zhao, Rune Bai, Fengming Yan, Caiyan Lei

**Affiliations:** College of Plant Protection, Henan Agricultural University, Zhengzhou 450046, China; xsx13125272472@163.com (S.X.); shibaozheng95@163.com (B.S.); hnzmdmmz@163.com (M.M.); zhaochen06166@163.com (C.Z.); yxbre@163.com (R.B.); fmyan@henau.edu.cn (F.Y.)

**Keywords:** B-box transcription factor, reactive oxygen species, resistance, cucumber, *Bemisia tabaci*

## Abstract

B-box (BBX) transcription factors play crucial roles in plant growth, development, and defense responses to biotic and abiotic stresses. In this study, we cloned a BBX transcription factor gene, *CsCOL9I,* from cucumber and analyzed its role in the plant’s defense against the feeding of *Bemisia tabaci*. *CsCOL9* is expressed throughout all developmental stages in cucumber, with the highest expression in the leaves. *CsCOL9* is induced by *B. tabaci* feeding, salicylic acid (SA), methyl jasmonate (MeJA), and hydrogen peroxide (H_2_O_2_). Cucumber plants with *CsCOL9* silence (TRV2-CsCOL9) and overexpression (1301-CsCOL9) were obtained and analyzed. After *CsCOL9* silencing, survival rates and host selectivity for *B. tabaci* increased; however, the expression levels of genes encoding enzymes (*CsSOD*, *CsRBOH*, *CsPOD*), activities of superoxide dismutase (SOD) and peroxidase (POD), and content of H_2_O_2_ in plants were all reduced. *CsCOL9* overexpression led to decreased survival rates and host selectivity for *B. tabaci*. Conversely, the expression levels of genes (*CsSOD*, *CsRBOH* and *CsPOD*), activities of SOD and POD, and content of H_2_O_2_ increased after *CsCOL9* overexpression in plants. Collectively, our results demonstrate *CsCOL9* positively regulates cucumber resistance to *B. tabaci* by activating reactive oxygen species bursts. This study lays a theoretical foundation for the application of *CsCOL9* in cucumber resistance breeding and green pest control of *B. tabaci*.

## 1. Introduction

Since the 1980s, *Bemisia tabaci*, (Hemiptera: Aleyrodidae) has spread globally due to trade activities involving ornamental plants like fuchsia and various economic crop seedlings, leading to widespread outbreaks [1,2]. In addition to directly feeding on plants, *B. tabaci* serves as a significant vector for plant viruses, capable of transmitting over 600 different types of plant viruses, which results in billions of dollars in economic losses each year [3]. However, chemical control methods have proven ineffective and environmentally damaging due to the pest’s high reproductive capacity and rapid development of resistance. Therefore, there is an urgent need for sustainable and effective control strategies.

Transcription factors (TFs), also known as trans-acting factors, specifically interact with cis-regulatory elements of eukaryotic genes to either activate or inhibit gene transcription. B-box (BBX) transcription factors represent a subclass of zinc finger proteins characterized by the presence of one single B-box domain or two arranged in tandem; some BBXs also contain CONSTANS (CO), CONSTANS-like (COL), and TIMING OF CAB EXPRESSION 1 (TOC1) domains, collectively referred to as the CCT domain [4,5,6,7]. In addition to participating in photomorphogenesis, photoperiod regulation, shade response, and other plant growth and development processes, BBX proteins also play important roles in various stress-induced plant defense responses [4,8,9,10]. Numerous studies have demonstrated that BBX proteins can enhance plant resistance by regulating the reactive oxygen species (ROS) system. For instance, sweet potato transcription factor *IbBBX24* protects plants from oxidative damage by mediating ROS clearance through the regulation of class III peroxidase gene *PRX17* [11]; overexpression of BBX from *Malus domestica* (*MdBBX1*) increases resistance to salt and drought in *Arabidopsis* by boosting the ROS scavenging system and alleviating oxidative stress [12]; overexpression of *MdBBX10* in *Arabidopsis* increases the plant’s ability to clear ROS, thereby enhancing drought resistance of transgenic *Arabidopsis* [13]; overexpression of *AtBBX29* improves the antioxidant capacity of sugarcane by reducing ROS accumulation and minimizing oxidative damage [14]; following overexpression of *SlBBX17*, transgenic tomato exhibits higher antioxidant enzyme activity and lower ROS levels, thus improving plants’ tolerance to high-temperature stress [15]; *CCT1* from the *Arabidopsis* BBX family promotes ROS accumulation by regulating defense signals induced by the bacterial flagellin protein flg22, enhancing *Arabidopsis* resistance to *Pseudomonas syringae* pv. DC3000 [16].

BBX proteins have increasingly been shown to regulate plant resistance against stress by modulating ROS levels. However, it remains unclear whether BBXs can influence ROS levels in host plants during damage caused by *B. tabaci*, thereby affecting the host’s resistance to this pest. In our transcriptomic analysis, we identified a gene (*CsaV3-609750*) that exhibited significant changes in expression levels following *B. tabaci* feeding and was predicted to encode a transcription factor [17]. To investigate the function and mechanism of this transcription factor in plant defense against *B. tabaci*, we cloned this gene from cucumber and analyzed its sequence and expression patterns. Then, we employed virus-induced gene silencing (VIGS) technology and gene overexpression techniques to manipulate the expression levels of the *COL9* gene in cucumber. Subsequently, we assessed the survival rates and free-choice behavior of *B. tabaci* on plants with either upregulated or downregulated *CsCOL9* expression. This study aims to explore the role of *CsCOL9* in enhancing plant defense responses against *B. tabaci*.

## 2. Results

### 2.1. Clone and Bioinformatics Analysis of CsCOL9 Gene

**Figure 1:**
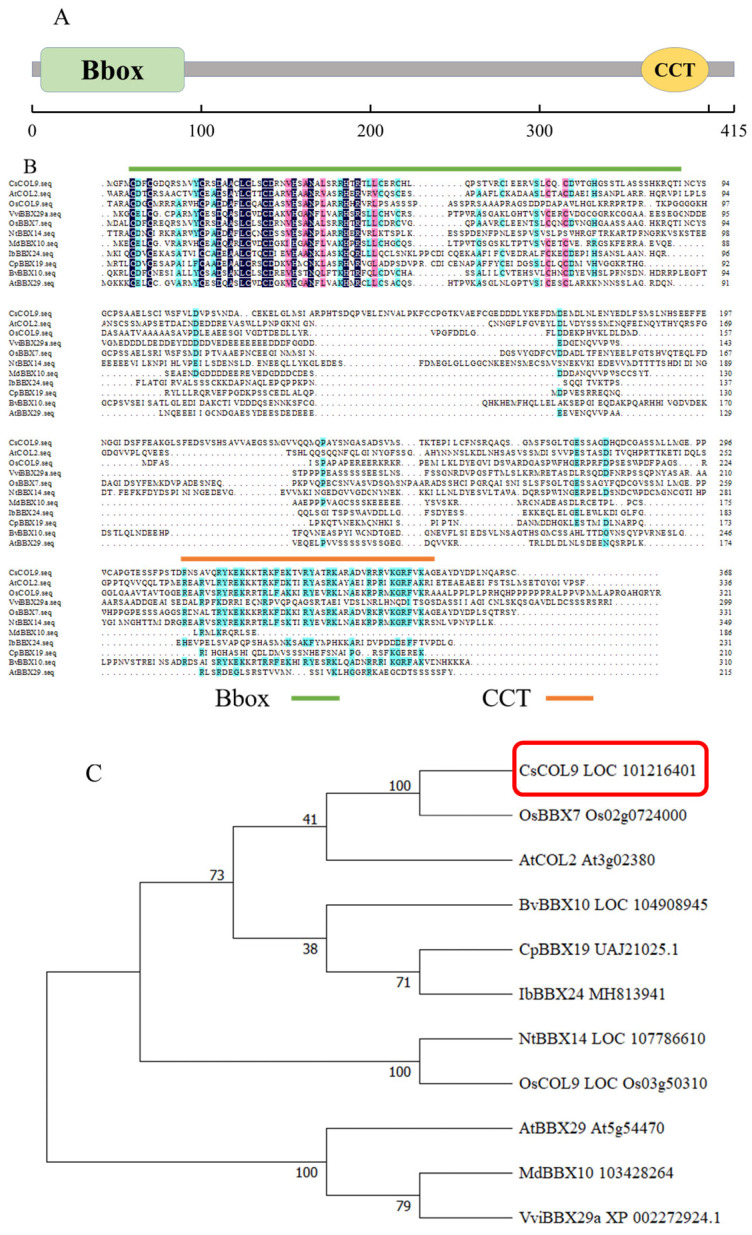
Bioinformatics analysis of *CsCOL9*. (**A**) Structure and domain composition of *CsCOL9*. The numbers indicate the amino acid positions of the corresponding conserved domains. Green and yellow rectangles represent the B-box and CCT domains, respectively. The scale bar represents 100 amino acids. (**B**) Multiple-sequence alignment of the *CsCOL9* protein with other typical BBX protein members. Green indicates B-box motif, orange indicates CCT motif). The analysis included the following proteins: *AtCOL2* (At3g02380), *OsCOL9* (LOC_Os03g50310), *VviBBX29a* (XP_002272924.1), *OsBBX7* (Os02g0724000), *NtBBX14* (LOC107786610), *MdBBX10* (103428264), *IbBBX24* (MH813941), *CpBBX19* (UAJ21025.1), *BvBBX10* (LOC104908945), and *AtBBX29* (At5g54470). Identical and similar amino acids are shaded, while highly conserved subdomains are indicated by horizontal lines. (**C**) Phylogenetic analysis of BBX proteins from cucumber (*CsCOL9*), rice (*OsBBX7* and *OsCOL9*), *Arabidopsis* (*AtCOL2* and *AtBBX29*), beetroot (*BvBBX10*), wintersweet (*CpBBX19*), sweet potato (*IbBBX24*), tobacco (*NtBBX14*), apple (*MdBBX10*), and grapevine (*VviBBX29a*). The phylogenetic tree was constructed using the neighbor-joining method in MEGA11.0, with numbers on nodes representing bootstrap support values. Red box indicates that it is the gene cloned in this article.

Based on transcriptome data [17], this study cloned a gene from cucumber that exhibited significant changes in expression level during feeding by *B. tabaci* and named it *CsCOL9*. The *CsCOL9* gene contains an open reading frame of 1248 bp (Table S2), encoding a protein composed of 415 amino acids (Table S3). *CsCOL9* contains an 87 aa B-box structural domain and a 44 aa transcriptional regulatory domain CCT (Figure 1A), classified as a B-box family zinc finger protein.

BLASTX analysis indicated that *CsCOL9* shares high homology with *CmCOL9* (XM_008465929.3) and *BhCOL9* (XM_039045922.1), with homologies of 95.11% and 92.11%, respectively. The amino acid sequence of *CsCOL9* was compared with reported BBX proteins involved in plant defense responses: *AtCOL2* (At3g02380), *OsCOL9* (LOC_Os03g50310), *VviBBX29a* (XP_002272924.1), *OsBBX7* (Os02g0724000), *NtBBX14* (LOC107786610), *MdBBX10* (103428264), *IbBBX24* (MH813941), *CpBBX19* (UAJ21025.1), *BvBBX10* (LOC104908945), and *AtBBX29* (At5g54470). The results showed that *CsCOL9* has relatively high homology with *OsBBX7* at 53.37% (Figure 1B). Using MEGA11.0 software, a phylogenetic tree for *CsCOL9* was constructed using the neighbor-joining method, yielding results consistent with the homology analysis, as shown in Figure 1C.

### 2.2. Expression Pattern Analysis of CsCOL9

#### 2.2.1. Spatiotemporal Expression Analysis of *CsCOL9*

**Figure 2:**
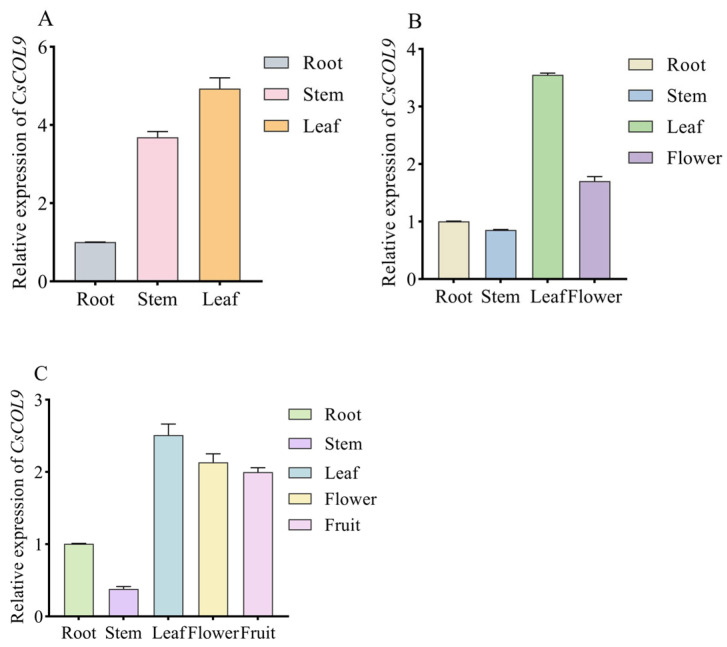
Expression analysis of *CsCOL9* in cucumber leaves at different developmental stages by RT-PCR. (**A**) Expression levels of *CsCOL9* in roots, stems, and leaves during the seedling stage. (**B**) expression levels of *CsCOL9* in roots, stems, leaves, and flowers during the flowering stage. (**C**) Expression levels of *CsCOL9* in roots, stems, leaves, flowers, and fruits during the maturity stage. The relative expression level of *CsCOL9* in roots is set to 1. Data are derived from three biological replicates, with the cucumber *ACTIN* gene used as an internal control to standardize expression levels. Averages and standard errors (SE) are represented by error bars. Asterisks indicate significant differences in gene expression based on *t*-tests.

To further explore the function of *CsCOL9*, this study examined its expression patterns using RT-qPCR. We assessed the expression levels of *CsCOL9* across different developmental stages and tissues of cucumber. The results revealed that the *CsCOL9* gene is expressed in various tissues throughout the entire development period of cucumber, with the highest levels found in leaves (Figure 2). Moreover, the expression level of *CsCOL9* in flowers increases over time, peaking at maturity, while its expression in stems declines as the cucumber develops.

#### 2.2.2. Expression of *CsCOL9* Was Induced by Exogenous Salicylic Acid, Methyl Jasmonate, Hydrogen Peroxide, and *B. tabaci* Feeding

**Figure 3:**
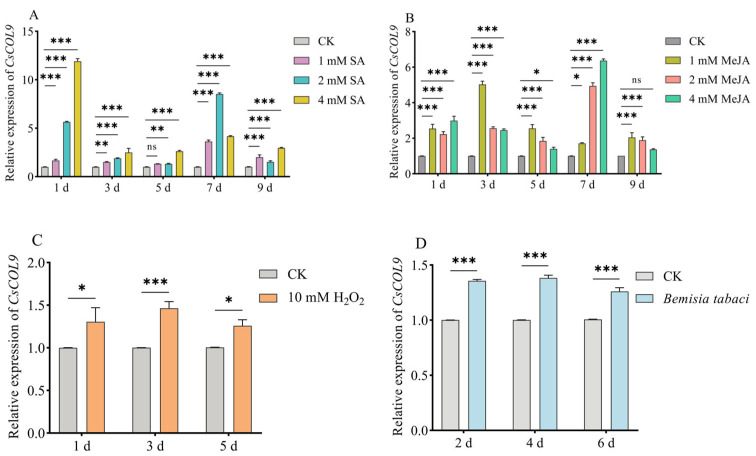
Expression analysis of *CsCOL9* under oxidative stress, plant hormone treatment and *B. tabaci* feeding. (**A**) Treatment with SA at concentrations of 0 mM, 1 mM, 2 mM, and 4 mM. (**B**) Treatment with MeJA at concentrations of 0 mM, 1 mM, 2 mM, and 4 mM. (**C**) Treatment with H_2_O_2_ at concentrations of 0 and 10 mM. (**D**) *B. tabaci* feeding. Data are derived from four biological replicates of cucumbers, with the *ACTIN* gene used as an internal control to standardize expression levels. Averages and standard errors (SE) are represented by error bars. Asterisks indicate significant differences in gene expression based on *t*-tests (ns, *p* > 0.05; * *p* ≤ 0.05; ** *p* ≤ 0.01; *** *p* ≤ 0.001).

This study examined the effects of stress treatments on the expression levels of *CsCOL9* in cucumber plants by using RT-qPCR. The results demonstrated that following the exogenous application of salicylic acid (SA), the expression level of *CsCOL9* was significantly upregulated, with the highest transcription level observed on the first day after spraying with 4 mM salicylic acid (Figure 3A). Similarly, after applying methyl jasmonate (MeJA), *CsCOL9* expression also increased, peaking on the 7th day post-application of 4 mM MeJA (Figure 3B). After hydrogen peroxide (H_2_O_2_) treatment, *CsCOL9* expression rose as well, reaching its maximum on the third day after treatment (Figure 3C). Furthermore, following *B. tabaci* inoculation, *CsCOL9* expression was also elevated, with the highest transcription level recorded on the 4th day after feeding (Figure 3D).

These findings indicate that exogenous treatment with SA, MeJA, H_2_O_2_, and *B. tabaci* feeding all activate the expression of *CsCOL9* in cucumbers, suggesting that *CsCOL9* plays a role in the plant’s defense response.

### 2.3. ROS Production Accumulated in Cucumber Seedlings Fed by B. tabaci

**Figure 4:**
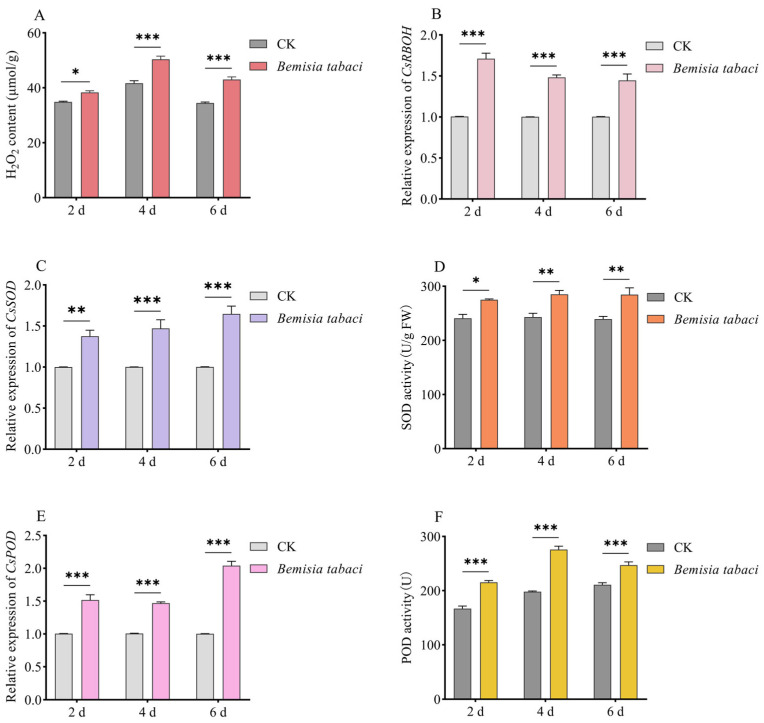
Effects of *B. tabaci* Feeding on ROS Generation in Cucumbers. (**A**) H_2_O_2_ content in cucumbers infested with *B. tabaci*. (**B**) Relative expression level of *CsRBOH*. (**C**) Relative expression level of *CsSOD*. (**D**) Activity of SOD. (**E**) Relative expression level of *CsPOD*. (**F**) Activity of POD; data are derived from four biological replicates of cucumbers, with the *actin* gene used as an internal control to standardize expression levels. Averages and standard errors (SE) are represented by error bars. Asterisks indicate significant differences in gene expression based on *t*-tests (* *p* ≤ 0.05; ** *p* ≤ 0.01; *** *p* ≤ 0.001).

To investigate whether *B. tabaci* feeding regulates ROS production in cucumbers, we compared the H_2_O_2_ contents of healthy cucumber plants with those of cucumber plants infested by *B. tabaci*. The results demonstrated that *B. tabaci* feeding resulted in a significant accumulation of H_2_O_2_ in cucumber leaves, exhibiting an increasing trend from day 2 to day 4 (Figure 4A). To further evaluate the impact of *B. tabaci* feeding on ROS in host plants, this study analyzed the expression levels of genes associated with ROS synthesis as well as their enzyme activities. The analysis revealed that following *B. tabaci* feeding, both *CsRBOH* and *CsSOD* were upregulated, with *CsRBOH* displaying the most pronounced increase on day 2 (Figure 4B) and *CsSOD* peaking on day 6 (Figure 4C). The activity of superoxide dismutase (SOD) significantly increased after *B. tabaci* feeding, peaking on day 4 (Figure 4D). After *B. tabaci* feeding, the relative expression level of *CsPOD* was upregulated, with the most notable increase occurring on day 6 (Figure 4E). Moreover, the activity of peroxidase (POD) differed between infested and healthy plants; infested plants exhibited higher POD activity at all three time points measured, peaking on day 4 (Figure 4F). These findings suggest that feeding by *B. tabaci* promotes ROS production and enhances the antioxidant capacity of host plants.

### 2.4. Silence of CsCOL9 Inhibited ROS Production in Cucumbers, Meanwhile Increased B. tabaci Survival and Host Preference

**Figure 5:**
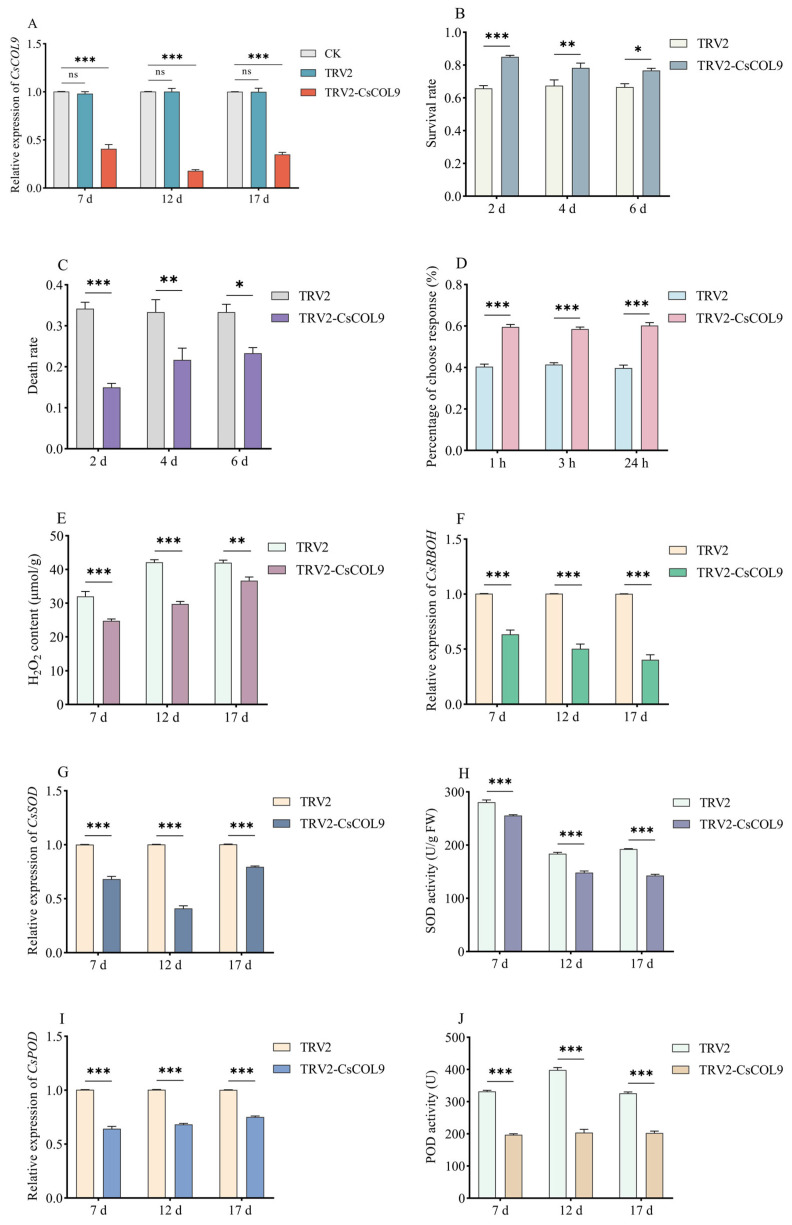
*CsCOL9* silence detection and its effects on *B. tabaci* and ROS. (**A**) *CsCOL9* silencing efficiency detection. (**B**) Survival rate of *B. tabaci*. (**C**) Mortality rate of *B. tabaci*. (**D**) Free choice of *B. tabaci*. (**E**) H_2_O_2_ content. (**F**) Relative expression levels of *CsRBOH*. (**G**) Relative expression levels of *CsSOD*. (**H**) SOD activity. (**I**) Relative expression levels of *CsPOD*. (**J**) POD activity. CK: Healthy plants. TRV2: Plants transformed with the empty vector. TRV2-CsCOL9: Plants transformed with the *CsCOL9* silencing vector. Data are derived from four biological replicates of cucumbers, with the *actin* gene used as an internal control to normalize expression levels. Averages and standard errors (SE) are represented by error bars. Asterisks indicate significant differences in gene expression based on *t*-tests (ns, *p* > 0.05; * *p* ≤ 0.05; ** *p* ≤ 0.01; *** *p* ≤ 0.001).

To explore the effects of *CsCOL9* on the defense response of host plant to *B. tabaci* feeding, we constructed a gene silence expression plasmid pNC-TRV2-COL9 based on virus-mediated gene silencing (VIGS) and transformed the plasmid into cucumber plants by *Agrobacterium* GV3101 injection. RT-qPCR results indicated that there was no significant difference in *CsCOL9* expression levels between plants transformed with the empty vector (TRV2) and those in the control group; however, the expression level of *CsCOL9* in TRV2-CsCOL9 plants was significantly downregulated (Figure 5A).

After obtaining *CsCOL9* gene-silenced cucumber seedlings (TRV2-CsCOL9), we analyzed ROS production in the plants and the survival and host selection changes of *B. tabaci* maintained in them. The results indicated that the survival rate of *B. tabaci* increased (Figure 5B), while the mortality rate of *B. tabaci* decreased as the expression level of *CsCOL9* in host plant declined (Figure 5C). Additionally, the number of *B. tabaci* landing on plants with *CsCOL9* silence significantly increased (Figure 5D). These results suggest that the silencing of *CsCOL9* in host cucumbers leads to an increase in both *B. tabaci* survival and its host preference.

ROS is important signal for inducing plant defense responses and was induced by *B. tabaci* feeding (Figure 4A), so we assessed the accumulation of H_2_O_2_ in control (TRV2) and *CsCOL9*-silenced cucumber plants (TRV2-CsCOL9) to explore whether *CsCOL9* silence would regulate H_2_O_2_ accumulation. Compared to the control, the level of H_2_O_2_ decreased in cucumbers with *CsCOL9* silence (Figure 5E), indicating that silencing of the *CsCOL9* gene leads to a reduction in H_2_O_2_ levels in *B. tabaci* host plants. The expression levels of *CsRBOH* and *CsSOD* were significantly lower in *CsCOL9*-silenced plants (TRV2-CsCOL9) compared to the control group (Figure 5F,G). Compared to the control group, SOD activity was significantly reduced in *CsCOL9*-silenced plants (Figure 5H). Following the decrease in *CsCOL9* expression level, there was also a corresponding decline in *CsPOD* expression level (Figure 5I) and POD activity (Figure 5J).

### 2.5. Overexpression of CsCOL9 in Host-Activated ROS Burst, Meanwhile, Reduced B. tabaci Survival and Its Host Preference

**Figure 6:**
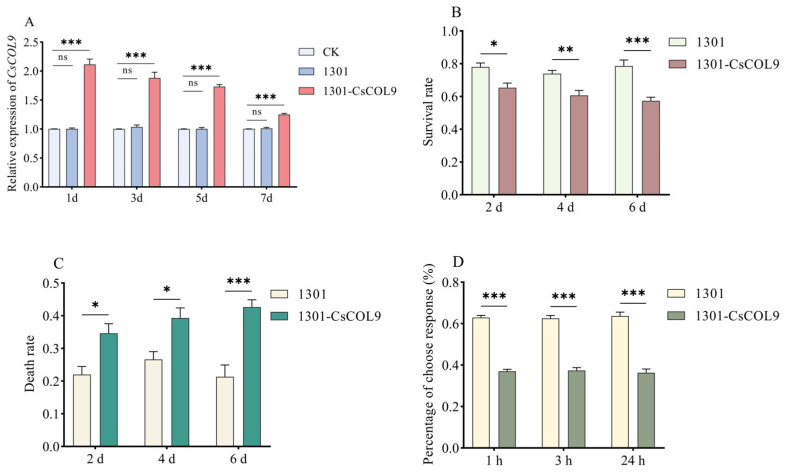

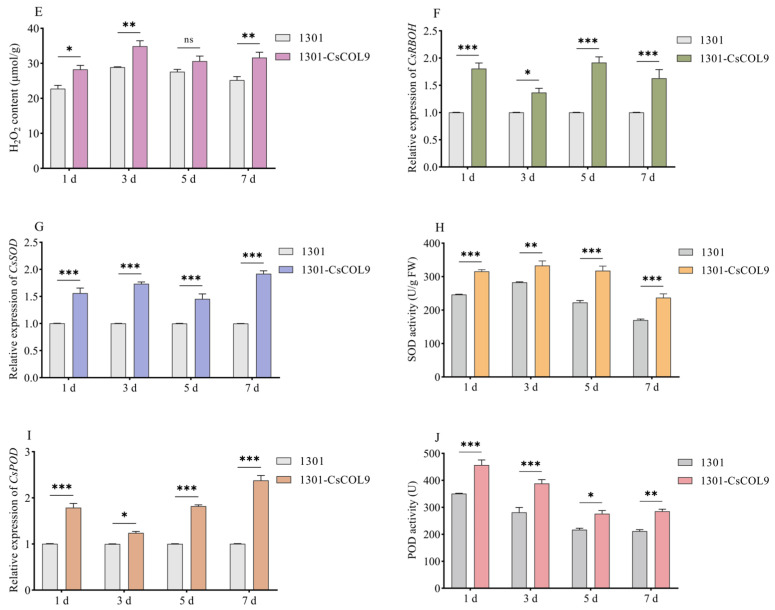
Detection of *CsCOL9* overexpression efficiency and its effects on *B. tabaci* and ROS. (**A**) *CsCOL9* overexpression efficiency detection. (**B**) Survival rate of *B. tabaci*. (**C**) Mortality rate of *B. tabaci*. (**D**) Free choice behavior of *B. tabaci*. (**E**) H_2_O_2_ contents. (**F**) Relative expression level of *CsRBOH*. (**G**) Relative expression level of *CsSOD*. (**H**) SOD activity. (**I**) Relative expression level of *CsPOD*. (**J**) POD activity. CK: Healthy cucumber. 1301: cucumber transformed with the empty vector pCAMBIA1301. 1301-CsCOL9: plants transformed with the *CsCOL9* overexpression vector. Data are derived from four biological replicates of cucumbers, with the *actin* gene used as an internal control to normalize expression levels. Averages and standard errors (SE) are represented by error bars. Asterisks indicate significant differences in gene expression based on *t*-tests (ns, *p* > 0.05; * *p* ≤ 0.05; ** *p* ≤ 0.01; *** *p* ≤ 0.001).

*CsCOL9* overexpression plasmid pCAMBIA1301-COL9 and empty vector pCAMBIA1301 were transformed in cucumber plants via *Agrobacterium*-mediated transformation. Quantitative PCR analysis showed there was no significant difference in *CsCOL9* expression levels between plants transformed with the empty vector (pCAMBIA1301) and the control group. However, in plants transformed with the 1301-CsCOL9, the expression level of *CsCOL9* significantly increased (Figure 6A).

To investigate whether overexpression of *CsCOL9* in host plants affects *B. tabaci*, we compared the survival rates and landing numbers of *B. tabaci* on *CsCOL9* overexpression cucumbers plants (1301-CsCOL9) to those on control plants (1301). The results showed that the survival rate of *B. tabaci* on the leaves of 1301-CsCOL9 plants decreased (Figure 6B), while the mortality rate increased (Figure 6C). Additionally, compared to cucumbers containing the empty vector (1301), the number of *B. tabaci* landing on 1301-CsCOL9 plants was significantly reduced (Figure 6D). These results indicate that increase of *CsCOL9* expression levels in host plants reduced *B. tabaci* survival rates and its host preference.

With the increased expression level of Cs*COL9*, the levels of H_2_O_2_ were significantly higher than those in the control group (Figure 6E). The expression levels of ROS metabolism-related genes *CsRBOH*, *CsSOD*, and *CsPOD* also increased with rising endogenous *COL9* expression (Figure 6F,G,I). Similarly, the activity of SOD and POD increased in seedlings with overexpression level of *CsCOL9* (Figure 6H,J). These results suggested that *CsCOL9* enhances host plant resistance to *B. tabaci* by regulating ROS signaling pathways.

## 3. Discussion

BBX proteins are a family of zinc finger transcription factors. More and more BBX genes are identified from different plant species. For example, a total of 536 *BBX* genes including 32 *AtBBXs*, 66 *BnaBBXs*, 81 *CsBBXs*, and 102 *BcBBXs* were identified from nine Brassicaceae species [18]. The BBX gene family can be divided into five structural groups depending on number of B-box domains and CCT domain [19]. Gene sequence analysis showed that *CsCOL9* has one B-box structural domain and one CCT domain (Figure 1A), therefore *CsCOL9* belongs to group III BBX proteins.

BBX proteins play crucial roles in regulating various plant growth and developmental processes, including seedling photomorphogenesis, flowering photoperiod regulation, and shade avoidance [4,5,7]. Our gene spatiotemporal expression patterns analysis revealed that *CsCOL9* is expressed in various parts of cucumber plants during the seedling, flowering, and fruiting stages (Figure 2A–C), indicating that *CsCOL9* may be involved in regulating the growth and development of cucumber leaves, flowers, and fruits.

Recent evidence suggests that BBX proteins play a role in plant defense responses. For instance, following infection by *Colletotrichum musae* in bananas, the expression level of the BBX protein gene (*MaCOL1*) increased, thereby enhancing the plant’s tolerance to biotic stress [20]. *IbBBX24* has been shown to promote jasmonic acid pathways in sweet potatoes, which enhances plant resistance to wilt disease [10]. The expression of *OsCOL9* was increased after *Magnaporthe oryzae* inoculation, and *OsCOL9* overexpression in transgenic rice plants showed increased blast resistance [21]. Similarly, our study demonstrated that feeding of *B. tabaci*, along with exogenous treatments of SA, MeJA, and H_2_O_2_, all activated *CsCOL9* expression in cucumber seedlings (Figure 3A–D). This indicates that *CsCOL9* is involved in the plant’s defense response.

Plant immune responses are extremely complex biological processes involving the generation of ROS, transcriptional regulation, programmed cell death, and production of secondary metabolites. Plants utilize these immune responses to recognize invading organisms (such as viruses, bacteria, fungi, oomycetes, nematodes, and insects) and activates their immune system for self-protection [22,23]. ROS serve as key regulators in plant defense responses and play significant roles in immune regulation [24,25]. When plants are damaged by insects, the ROS accumulated not only promote lignification within plant tissues, forming a direct physical barrier and enhancing defense capabilities [26], but also induce the synthesis of defense enzymes through relevant defense genes. *Pinus sylvestris* responds to insect egg deposition by ROS accumulation linked with reduced activity of the ROS scavenger catalase [27]. H_2_O_2_ is the most stable ROS, which can penetrate cell membranes in molecular form and also serve as an intracellular signal to activate defense responses [28,29]. Increasing evidence highlights that ROS, particularly H_2_O_2_, play significant roles in plant responses to herbivorous insects feeding [30,31,32]. For example, H_2_O_2_ could mitigate damage from *Ostrinia nubilalis* to transgenic maize [30]. H_2_O_2_ accumulation was induced by insect feeding in several reports; for instance, the larvae of *Mayetiola destructor* can rapidly induce H_2_O_2_ accumulation at damaged sites in wheat [33]; feeding by *Acyrthosiphon pisum* increases both H_2_O_2_ and superoxide anion levels in pea leaves, enhancing plant resistance against this aphid [34]; H_2_O_2_ levels in strawberry increased to generate responses to the damage caused by *Tetranychus urticae* [35]. Similarly, our study found that feeding by *B. tabaci* increased H_2_O_2_ levels in host plants; specifically, on day 4 post-inoculation with *B. tabaci*, H_2_O_2_ content rose by 24.771% (Figure 4A).

Activity changes in defense enzymes are another important aspect of plant responses to insect feeding [36,37]. Activities of ROS scavenging enzymes in lima bean leaves were enhanced when fed by *Spodoptera frugiperda*, thereby reducing oxidative damage caused by their feeding behavior [38]. Activities of SOD and POD in host plants increased after infestations by *Grapholita molesta* combined with *Cydia pomonella* [39]. After being damaged by *Manduca sexta*, the transcription level of NADPH oxidase gene *NaRBOHD* in tobacco plants was rapidly induced. In addition, the silencing of the *NaRBOHD* gene reduced ROS accumulation, making tobacco more susceptible to pests [40]. In our study, gene expression levels of *CsRBOH*, *CsSOD* and *CsPOD*, as well as activities of SOD and POD in host plants increased after feeding by *B. tabaci* (Figure 4B–F), indicating a potential role for ROS in the defensive processes activated against attacks from *B. tabaci*.

Increasing studies have shown that BBX transcription factors can regulate plant resistance to stress by regulating plant ROS systems. Overexpression of BBX transcription factors could reduce the content of ROS levels in plants, thereby enhancing their adaptability to salt, drought, oxidative stress, and high-temperature stress [11,12,13,14,15]. There are also studies reporting that BBX transcription factors increase plant resistance to fungal pathogens and piercing–sucking pests by promoting ROS accumulation [16,41]. This variability may be due to the different roles of ROS in plants responding to various stresses. In our study, silencing of *CsCOL9* in cucumber plants resulted in decreased resistance against *B. tabaci*, as evidenced by increased survival rates and host preference of *B. tabaci* (Figure 5B,D). Conversely, overexpression of *CsCOL9* improved the plant’s resistance to *B. tabaci*, reflected by reduced survival rates and preference of *B. tabaci*, (Figure 6B,D). These findings indicate that *CsCOL9* positively regulates host plant resistance against *B. tabaci*. Further research found that contents of H_2_O_2_, expression levels of antioxidant enzyme genes (*CsRBOH*, *CsSOD*, *CsPOD*), activities of antioxidant enzymes (SOD, POD) in host plants decreased after *CsCOL9* gene silencing (Figure 5F–I) and increased after *CsCOL9* gene overexpression (Figure 6F–I). Our study proved that *CsCOL9* enhances the resistance of host plants to *B. tabaci* by positively regulating the burst of ROS in these plants. Our findings offer new theoretical insights into how BBX proteins function biologically under stress, with significant implications for cucumber breeding efforts aimed at achieving greater resilience in the future.

## 4. Materials and Methods

### 4.1. Plant and Insect

*Cucumis sativus* (var. Bojie-18A) (Derit Seeds Biotechnology Co., Ltd., Tianjin, China) cucumber plants were cultivated in plastic pots (10 cm in diameter and 12 cm in height), with one plant per pot, under controlled conditions (photoperiod 16L:8D; a constant temperature of 26 °C; humidity 40%) in a greenhouse at Henan Agricultural University, Zhengzhou, China.

A colony of *B. tabaci* biotype Q (Mediterranean, MED) was maintained on healthy cucumber plants. The genetic purity of the *B. tabaci* biotype Q was monitored every three generations using the random amplified polymorphic DNA polymerase chain reaction (RAPD-PCR) technique, combined with sequencing of the *mtCO1* gene [42].

The tested strains of *Escherichia coli* (DH5α) and *Agrobacterium tumefaciens* (GV3101) were obtained from Beijing Qinke Biotechnology Co., Ltd., Beijing, China. The VIGS silencing vectors (pNC-TRV1 and pNC-TRV2) were provided by Professor Shi Yan’s research group at Henan Agricultural University.

### 4.2. Gene Expression Pattern Analysis

For analysis of gene spatiotemporal expression patterns, cucumber plants exhibiting consistent growth were selected. Samples of roots, stems, and leaves at the seedling stage; roots, stems, leaves, flowers at the flowering stage; and roots, stems, leaves, flowers, and fruits at the mature stage were collected. RNA was extracted from these samples for real-time quantitative PCR (RT-qPCR), with the relative expression level of *CsCOL9* in the roots set to 1 to analyze its expression patterns across different developmental stages and plant parts. Each sample part at each stage included four biological replicates.

Two leaf cucumber seedlings with similar growth were subjected to various treatments. For salicylic acid (SA) (0 mM, 1 mM, 2 mM, 4 mM), methyl jasmonate (MeJA) (0 mM, 1 mM, 2 mM, 4 mM), and hydrogen peroxide (H_2_O_2_) (0 mM, 10 mM) treatments, seedlings were sprayed with different concentrations of solutions. Samples were collected on days 1, 3, 5, 7, and 9 after treatment and subsequently frozen in liquid nitrogen for RT-qPCR analysis. Control plants were sprayed with water. Each time point included four biological replicates.

For *B. tabaci* treatment, each cucumber plant was inoculated with 30 *B. tabaci* adults using a micro-insect clip cage; healthy cucumbers served as controls without insect inoculation. Samples were taken on days 2, 4, and 6 post-inoculation and frozen in liquid nitrogen for subsequent analysis of qPCR results as well as enzyme activity and endogenous levels of hydrogen peroxide. Each time point included four biological replicates.

### 4.3. Gene Isolation and Sequence Analysis

Total RNA was extracted from cucumber samples using the RNAiso Plus reagent kit (Takara, San Jose, CA, USA), and first-strand cDNA synthesis was conducted according to the protocol provided by the PrimeScript™ RT Reagent Kit with gDNA Eraser (Perfect Real Time) (Takara, USA). High-fidelity enzymes and specific primers (Table S1) were employed to clone *CsCOL9* from the cDNA. The cloned *CsCOL9* was then ligated into the pMD18-T vector and transformed into *Escherichia coli* DH5α. Positive clones were identified and sequenced.

The *CsCOL9* sequence was uploaded to the NCBI database’s Protein BLAST for homology analysis, and conserved domains of *CsCOL9* were examined through the NCBI database. Additionally, amino acid sequence alignment of *CsCOL9* was performed using DNAMAN software, version 9.

### 4.4. RT-qPCR Analysis

RT-qPCR analysis was conducted using the SYBR Green method with the TB Green^®^ Premix Ex Taq™ II (Tli RNaseH Plus) (Takara, San Jose, CA, USA). Total RNA was extracted using the RNAiso Plus reagent kit. According to the instructions of the reverse PrimeScript™ RT Reagent Kit with gDNA Eraser (Perfect Real Time) (Takara, USA), total RNA (total RNA = 1 μg) was reverse-transcribed into cDNA, and then quantitative fluorescence detection was performed [43]. The housekeeping gene *actin* in cucumber plants served as an endogenous control in the RT-qPCR studies to analyze the transcription levels of *CsCOL9*, *CsRBOH*, *CsSOD*, and *CsPOD*. Primers used in RT-qPCR are shown in Table S1 with *actin* used as an internal control to normalize expression levels. Each time point included four biological replicates.

### 4.5. Determination of Enzymes Activities and Hydrogen Peroxide Contents

A 0.1 g quantity of true leaves with the veins removed was placed in a 1.5 mL enzyme-free centrifuge tube, and then frozen in liquid nitrogen. On ice, 0.1 g of leaf sample was ground in 1 mL of PBS buffer (pH 7.8, 1% PVP, 0.2 mM EDTA) to obtain a homogenate, which was then centrifuged at 12,000× *g* for 20 min at 4 °C. The supernatant was collected for enzyme activity assays [44]. Each time point included four biological replicates.

Determination of POD activity followed the method described by Zhen et al., measuring changes in absorbance at OD_470_ over time and calculating the enzyme reaction rate based on absorbance changes over a period of 120 s. Assay of SOD activity assay also referenced Zhen et al.’s methodology; samples were illuminated under 4000 lux at 25 °C for 15 min, with a control group containing only the buffer solution. Absorbance was measured at OD_560_, with a reduction of NBT photochemical reaction by 50% defined as one unit of enzyme activity [45,46]. H_2_O_2_ content was determined by using an H_2_O_2_ assay kit (Suzhou Grace Biotechnology Co., Ltd., Suzhou, China).

### 4.6. Generation of Cucumber with Silenced or Overexpressed CsCOL9 Gene

The full-length CDS of *CsCOL9* was amplified using primer pairs from the pMD18-T-COL9 plasmid and subsequently ligated into the pNC-TRV2 plasmid and the pCAMBIA1301 plasmid to construct the gene-silencing vector pNC-TRV2-COL9 and the overexpression vector pCAMBIA1301-COL9, respectively [47].

Recombinant plasmids pNC-TRV2-COL9 and the empty plasmid pNC-TRV2 were transformed into *Agrobacterium tumefaciens* strain GV3101. When the *Agrobacterium* reached an OD_600_ of 1.2 at 28 °C, they were mixed in equal proportions with pNC-TRV1 and injected into the cotyledons of cucumber seedlings with only one true leaf. Plant materials were collected on days 7, 12, and 17 after transformation for gene expression analysis, H_2_O_2_ content measurement, and oxidase activity assays.

Similarly, recombinant plasmid pCAMBIA1301-COL9 and the empty plasmid pCAMBIA1301 were transformed into *Agrobacterium tumefaciens* strain GV3101. After reaching an OD_600_ of 1.2 at 28 °C, engineering agrobacterium were injected into the cotyledons of cucumber seedlings with only one true leaf. Plant materials were collected on days 1, 3, 5, and 7 after transformation for gene expression analysis, H_2_O_2_ content measurement, and oxidase activity assays.

### 4.7. B. tabaci Bioassay

Twelve days after injection of engineering agrobacterium including silencing vector pNC-TRV2-COL9 and six days after injection of engineering agrobacterium including overexpression vector pCAMBIA1301-COL9, free choice experiments and mortality assay of *B. tabaci* were conducted. This experiment was carried out in the greenhouse of the College of Plant Protection at Henan Agricultural University (photoperiod 16L:8D; a constant temperature of 26 °C; humidity 40%).

For the free choice experiment, cucumber plants with the *CsCOL9* gene silenced (TRV2-CsCOL9) and control plants with empty vectors (TRV) were placed diagonally with insect rearing cages. One hundred *B. tabaci* adults were released between the two plants. The number of *B. tabaci* landing on each plant were counted at 1 h, 3 h, and 24 h post-release. The numbers of *B. tabaci* landing on *CsCOL9* gene overexpression plants (1301-COL9) and control plants (1301) were recorded by using the same method [48]. The experiment was repeated eight times.

For the *B. tabaci* mortality assay, every 30 adults were collected and placed in a micro-insect cage. These cages were then clipped onto the first true leaf of cucumber plants with *CsCOL9* gene silencing (TRV2-CsCOL9) and overexpression (1301-CsCOL9) separately. On days 2, 4, and 6, the numbers of live and dead *B. tabaci* within the micro-insect cages were recorded. Adults in cages clipped on plants with the empty gene silence vector (pNC-TRV2) and overexpression vector (pCAMBIA1301) were used as a control. The experiment was repeated eight times.

### 4.8. Data Analysis

All the experiments include three technical replicates. Experimental data were analyzed using Graphpad Prism 8.0.2 software. Two-way ANOVA was employed to analyze the differences between treatment groups and control groups in the experiments, with a significant level set at 0.05. The 2^−ΔΔCt^ method was used for numerical conversion and comparative analysis of RT-qPCR data. Results are presented as mean ± standard error (mean ± SE).

## Data Availability

The original contributions presented in the study are included in the article/Supplementary Material, further inquiry can be directed to the corresponding author.

## Supplementary Materials

The supporting information can be downloaded at: https://www.mdpi.com/article/10.3390/ijms26010324/s1.
