# Supplementary material for: A B-Box (BBX) Transcription Factor from Cucumber, CsCOL9 Positively Regulates Resistance of Host Plant to Bemisia tabaci
Source: Int J Mol Sci. 2025 Jan 2;26(1):324. doi: 10.3390/ijms26010324 (PMC11720035; doi:10.3390/ijms26010324)
Supplement: Supplementary file 1 [file ijms-26-00324-s001.zip › Table S1. Primer sequence.pdf]

	primer name	forward primer	reverse primer
1	COL9	ATGGGTTTCATGTGTGATTTTTGTG	TCAGCAGCTTCTAGCCTGG
2	VIGS-COL9	GGACAGGTCATGGCTCATCT	TCACTGGCTTCTGTTGACCC
3	VIGS-TRV2-COL9	AGTGGTCTCTGTCCAGTCCT<br>GGACAGGTCATGGCTCATCT	GGTCTCAGCAGACCACAAGT<br>TCACTGGCTTCTGTTGACCC
4	VIGS-TRV2	GGCGGTTCTTGTGTGTCAAC	CAAGATCAGTCGAGAATGTC
5	1301-COL9	CGGTACCCGGGGATCCTCTAGA<br>ATGGGTTTCATGTGTGATTTTTGTG	ACGGCCAGTGCCAAGCTT<br>TCAGCAGCTTCTAGCCTGGT
6	q-ACTIN	TCCACGAGACTACCTACAACTC	GCTCATACGGTCAGCGAT
7	q-RBOH	TTGCTGGGAAGAGTGGGT	GCTCCAATACCAAGACCAAC
8	q-SOD	GGAAAGATGTGAAGGCTGTGG	GCACCATGTTGTTTTCCAGCAG
9	q-POD	CTATGCCAAAAGCTGCCCAC	ACCCCTGACAAAGCAATCGT
10	q-AOS	CGGTGTTTGAGGAAGCGGATAG	TGTGGAGATTCACTCCGGCTC
11	q-LOX2	TGCTTGGCTAACCGACGAAG	TGCCTCAGCAACTGTAAGACC
12	q-MYC2	GCTCCAAATGGCAGAATCGG	CATCGCATCCCAACCCATGA
13	q-NPR1	TGGAGAGAGAGATGCGTAGG	ATTGCCACCTTCGCCTC
14	q-PR3	GGTCCTCCTCTCTATCGGTG	AGTGGCCTGGAATCCGAC
15	q-PAL	CAAAGCGCCGAGCAACAC	TTCCTCCAAATGCCTCAAGTC

Note: The red part indicates the homologous recombination sequence, and the primers labeled with "q" are quantitative fluorescent primers.
