# Supplementary material for: A B-Box (BBX) Transcription Factor from Cucumber, CsCOL9 Positively Regulates Resistance of Host Plant to Bemisia tabaci
Source: Int J Mol Sci. 2025 Jan 2;26(1):324. doi: 10.3390/ijms26010324 (PMC11720035; doi:10.3390/ijms26010324)
Supplement: Supplementary file 1 [file ijms-26-00324-s001.zip › Table S2. CsCOL9 gene sequence.pdf]

>XM_031887293.1:100-1347 PREDICTED: Cucumis sativus zinc finger protein CONSTANS-LIKE 9 (LOC101216401), transcript variant X1, mRNA

ATGGGTTTCATGTGTGATTTTTGTGGGGATCAGAGGTCAATGGTTTACTGCCGATCTGAT
GCTGCGTGTTTGTGCTTATCATGTGACCGTAATGTCCATTCTGCTAACGCCTTGTCGAGG
CGTCATACGAGAACACTGTTGTGTGAAAGATGCCATTTGCAACCCTCCACAGTGAGGT
GTATTGAAGAGAGAGTTTCTCTGTGTCAAAATTGTGATTGGACAGGTCATGGCTCATCT
ACCCTTGCTTCATCATCACACAAGAGGCAAACCATAAATTGTTACTCTGGTTGTCCATC
CGCTGCAGAACTTTCTTGCATCTGGTCCTTTGTGTTGGATGTTCCATCTGTAAATGATGC
TTGTGAGAAAGAGTTGGGATTGATGAGCATTGCTGAGACTGACTTGACGGGCGCCTGG
AGTCCTTCGGAGAACAACGCTGGTCAAAGAATGCCTGGGTCAACAGAAGCCAGTGAT
GTTTGTAGCAGGGAAAAGTCAAATGTTTTGGTTGGATCATCTTCACTCATTGGCTCTAG
GCCTCACACTTCAGATCAGCCAGTTGAATTGGATAATGTGGCTTTACCCAAGTTTTGCT
GTCCTGGAACAAAAGTTGCTGAATTCTGCGGTGAGGATGACGACCTCTACAAGGAGTT
TGATATGGATGAAATGGATTTGAATCTTGAAAATTATGAGGACCTATTTAGCATGAGCCT
TAATCATTCTGAAGAGTTTTTCGAGAATGGTGGAATTGATAGCTTTTTTGAGGCAAAAG
GCCTGTCTTTTGAAGATTCAGTTTCCCACAGTGCAGTTGTTGCAGAGGGTTCTTCCATG
GGAGTGGTCCAACAAATGCAGCCAGCTTACAGCAATGGTGCCTCTGCTGACTCAGTGA
TGAGTACTAAAACCGAACCTATTCTCTGTTTCAATTCAAGGCAAGCCCAGTCGGGTATG
TCCTTTTCTGGTCTTACTGGTGAGAGTAGTGCTGGTGATCATCAAGACTGTGGAGCCTC
TTCAATGCTTCTAATGGGTGAGCCTCCATGGTGTGCACCTGGCACTGAAAGTTCCTTCC
CATCTACTGATCGCAATAGTGCCGTTCAGCGTTATAAGGAGAAAAAGAAGACACGCAA
GTTTGAGAAAACAGTGAGATATGCCACTCGCAAAGCGAGAGCTGATGTCAGAAGGCG
TGTGAAGGGACGGTTTGTGAAGGCTGGAGAGGCATATGATTACGATCCACTAAACCAG
GCTAGAAGCTGCTGA
