# Supplementary material for: A B-Box (BBX) Transcription Factor from Cucumber, CsCOL9 Positively Regulates Resistance of Host Plant to Bemisia tabaci
Source: Int J Mol Sci. 2025 Jan 2;26(1):324. doi: 10.3390/ijms26010324 (PMC11720035; doi:10.3390/ijms26010324)
Supplement: Supplementary file 1 [file ijms-26-00324-s001.zip › Table S3. CsCOL9 protein sequence.pdf]

>ref|XP_031743153.1|:1-415 zinc finger protein CONSTANS-LIKE 9 [Cucumis sativus]

MGFMCDFCGDQRSMVYCRSDAACLCLSCDRNVHSANALSRRHTRTLLCERCHLQPSTV
RCIEERVSLCQNCDWTGHGSSTLASSSHKRQTINCYSGCPSAAELSCIWSFVLDVPSVNDA
CEKELGLMSIAETDLTGAWSPSENNAGQRMPGSTEASDVCSREKSNVLVGSSSLIGSRPH
TSDQPVELDNVALPKFCCPGTKVAEFCGEDDDLYKEFDMDEMDLNLENYEDLFSMSLNH
SEEFFENGGIDSFFEAKGLSFEDSVSHSAVVAEGSSMGVVQQMQPAYSNGASADSVMST
KTEPILCFNSRQAQSGMSFSGLTGESSAGDHQDCGASSMLLMGEPPWCAPGTESSFPSTD
RNSAVQRYKEKKKTRKFEKTVRYATRKARADVRRRVKGRFVKAGEAYDYDPLNQARS
C
